# Urinary and Sexual Function Outcomes After Ultrasound-Guided High-Intensity Focused Ultrasound for Uterine Fibroids: Associations of Lower Urinary Tract Symptoms with Fibroid Location and Uterine Morphology

**DOI:** 10.3390/biomedicines14102219

**Published:** 2026-10-01

**Authors:** Cheng-Yu Long, Cherng-Jye Jeng, Liang-Ying Ke

**Affiliations:** 1 Department of Obstetrics and Gynecology, Kaohsiung Medical University Hospital, Kaohsiung Medical University, Kaohsiung 80756, Taiwan; 830263@kmuh.org.tw (C.-Y.L.); jengcj@gmail.com (C.-J.J.); 2 Department of Obstetrics and Gynecology, College of Medicine, Kaohsiung Medical University, Kaohsiung 807378, Taiwan; 3 Department of Gynecology, Leo Minimally Invasive International Hospital, New Taipei City 244019, Taiwan; 4 Department of Obstetrics and Gynecology, Kaohsiung Municipal Siaogang Hospital, Kaohsiung Medical University, Kaohsiung 812, Taiwan

**Keywords:** uterine fibroids, high-intensity focused ultrasound, lower urinary tract symptoms, overactive bladder, uterine morphology

## Abstract

**Background/Objectives**: Uterine fibroids may contribute to lower urinary tract symptoms (LUTS) through pelvic mass effects, but urinary and sexual outcomes after ultrasound-guided high-intensity focused ultrasound (USgHIFU) and their anatomical correlates remain unclear. We evaluated 6-month urinary and sexual outcomes, described urinary changes by dominant fibroid location, and explored associations between uterine morphology and baseline urinary symptom burden. **Methods**: This retrospective study included 75 women with paired baseline and 6-month urinary assessments (anterior, *n* = 45; non-anterior, *n* = 30). Measures included the Overactive Bladder Symptom Score (OABSS), Urogenital Distress Inventory-6 (UDI-6), Incontinence Impact Questionnaire-7 (IIQ-7), International Consultation on Incontinence Questionnaire–Urinary Incontinence Short Form (ICIQ-UI SF), and Female Sexual Function Index (FSFI; *n* = 63); pretreatment magnetic resonance imaging (MRI) characterized uterine anatomy. **Results**: At 6 months, OABSS decreased from 3.51 ± 3.07 to 2.57 ± 2.29 (*p* = 0.005); UDI-6, IIQ-7, and ICIQ-UI SF also improved (all *p* ≤ 0.009), and OABSS ≥ 3 prevalence declined from 53.3% to 40.0% (*p* = 0.002). Mean urinary symptom scores decreased in both location groups. The larger longitudinal-diameter subgroup had higher baseline OABSS (4.20 vs. 2.71; *p* = 0.034) and OABSS ≥ 3 prevalence (70.0% vs. 34.3%; *p* = 0.003), whereas the corresponding comparisons for uterine volume and anteroposterior diameter were not significantly associated. The mean FSFI total score increased from 21.6 ± 5.8 to 26.5 ± 6.6 (summary-derived conservative *p*-value bound, *p* < 0.005). **Conclusions**: USgHIFU was associated with improved urinary symptoms, urinary symptom-related quality of life, and sexual function. The exploratory association between the longitudinal-diameter groups and baseline overactive bladder symptom burden warrants confirmation using continuous measurements and adjusted analyses.

## 1. Introduction

Uterine fibroids commonly cause abnormal uterine bleeding, pelvic pain, bulk-related symptoms, and lower urinary tract symptoms (LUTS), including frequency, urgency, nocturia, urinary incontinence, and voiding difficulty, which may impair quality of life [1].

Fibroid-associated LUTS are often attributed to bladder compression, and anterior fibroids have been presumed to cause more prominent symptoms. Parker-Autry et al. reported more severe voiding symptoms with dominant anterior fibroids and an association between greater uterine volume and urgency [2]. However, a nationwide study found that greater leiomyoma volume was associated with urinary incontinence but not frequent urination, while anterior location was not consistently associated with urinary symptoms [3]. Thus, LUTS may depend on symptom type, overall uterine configuration, and the uterus-bladder relationship rather than fibroid location alone.

Ultrasound-guided high-intensity focused ultrasound (USgHIFU) is a noninvasive, uterus-preserving treatment that induces fibroid coagulative necrosis followed by shrinkage and symptom relief. HIFU reduces fibroid volume and symptom severity and improves quality of life [4,5]. Our previous 500-case study evaluated urinary and sexual outcomes after HIFU but focused on overall effectiveness and safety rather than anatomical factors associated with urinary outcomes [5]. Sexual function may also improve after HIFU [6]. Urinary symptom profiles across fibroid locations and whole-uterus morphology warrant further characterization.

This study evaluated 6-month changes in LUTS and urinary symptom-related quality of life after USgHIFU, with exploratory descriptions of urinary changes according to dominant fibroid location. We also explored associations between baseline overactive bladder symptom burden and uterine volume, longitudinal diameter, and anteroposterior diameter. Female sexual function was assessed as a secondary outcome.

## 2. Materials and Methods

### 2.1. Study Design and Participants

**Figure 1:**
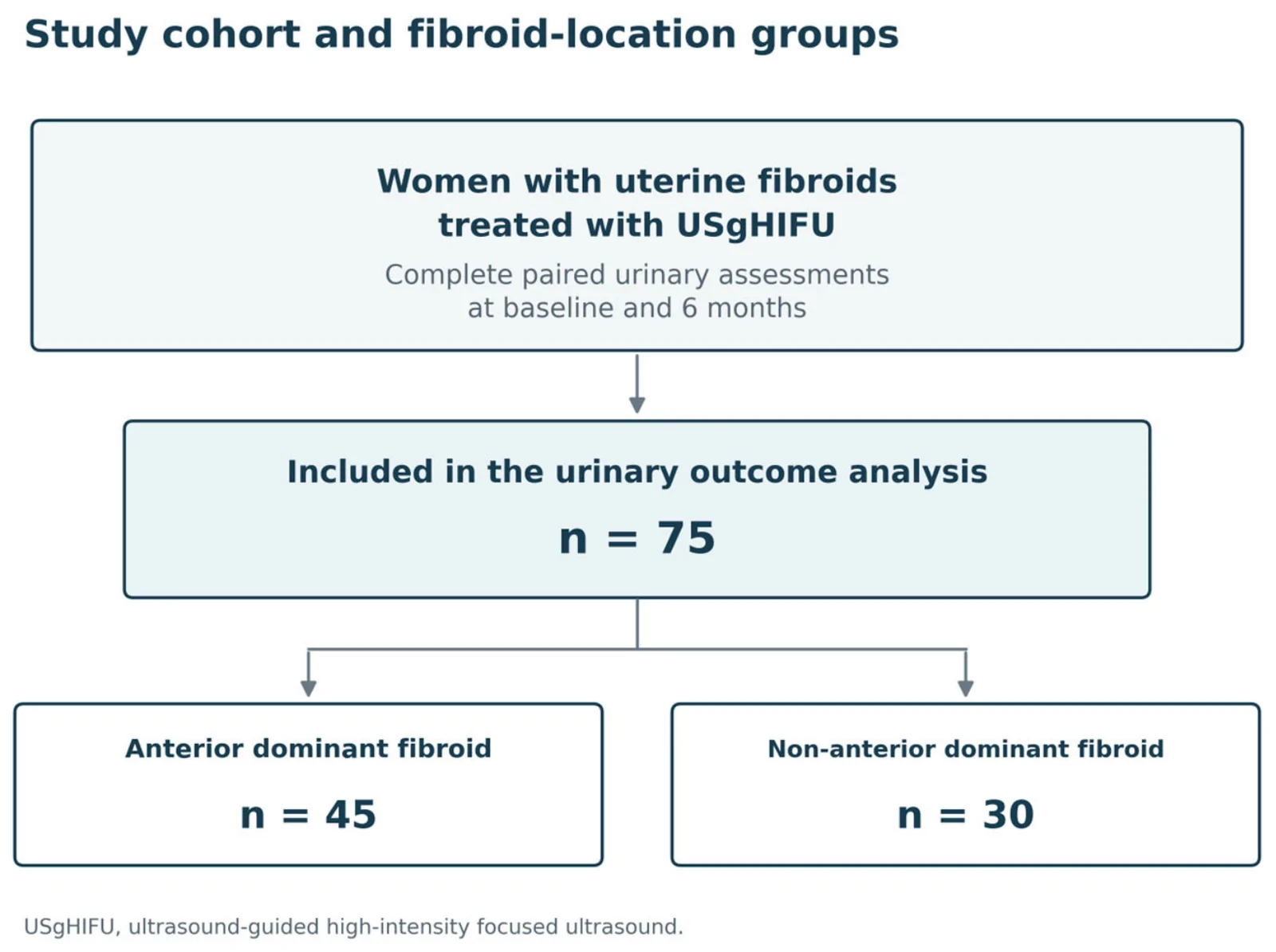
Study cohort and classification by dominant fibroid location. Women with uterine fibroids treated with ultrasound-guided high-intensity focused ultrasound (USgHIFU) who had complete urinary assessments at baseline and 6 months were included in the analysis (*n* = 75). Participants were classified according to dominant fibroid location into anterior (*n* = 45) and non-anterior (*n* = 30) groups.

This retrospective observational study included women who underwent USgHIFU for MRI-confirmed uterine fibroids at Kaohsiung Medical University Chung-Ho Memorial Hospital between April 2015 and June 2019 and had complete baseline and 6-month urinary assessments. Baseline and six months denote the outcome-assessment time points; the present investigation was designed as a retrospective review of existing clinical records. The requirement for paired urinary assessments defined the complete-case analytical cohort. Seventy-five women were included: 45 with an anterior and 30 with a non-anterior dominant fibroid. Complete paired Female Sexual Function Index (FSFI) assessments were available for 63 women. The remaining 12 women had complete paired urinary assessments but did not complete the FSFI questionnaires and were subsequently lost to further follow-up. These women were retained in the urinary analyses. The retrospective record-review protocol concerning HIFU outcomes in uterine fibroids and adenomyosis was approved by the Institutional Review Board of Kaohsiung Medical University Chung-Ho Memorial Hospital (KMUHIRB-E(II)-20200382; approved on 28 December 2020). The requirement for informed consent was waived. The approval concerned retrospective research use of existing records, distinct from the earlier dates of clinical treatment (Figure 1).

### 2.2. Clinical and Questionnaire Assessments

Age, body mass index, and uterine measurements were obtained from medical records. LUTS, urinary symptom-related quality of life, and sexual function were assessed at baseline and 6 months. The Overactive Bladder Symptom Score (OABSS; range, 0–15) evaluates daytime and nighttime frequency, urgency, and urgency urinary incontinence, with higher scores indicating greater severity [7]. OABSS ≥ 3 indicates greater symptom burden rather than a definitive clinical diagnosis.

Urogenital symptom distress and quality-of-life impact were evaluated using the Urogenital Distress Inventory-6 (UDI-6) and Incontinence Impact Questionnaire-7 (IIQ-7) [8]. Item sums were divided by the maximum possible score and multiplied by 100 to obtain standardized 0–100 scores. Urinary incontinence was assessed using the International Consultation on Incontinence Questionnaire–Urinary Incontinence Short Form (ICIQ-UI SF; range, 0–21) [9]. Higher scores indicated worse symptoms or impact. Accordingly, lower urinary questionnaire scores at follow-up were interpreted as improvement in the corresponding patient-reported outcomes. Changes were interpreted on the original score scales without applying a threshold for clinically important improvement. Sexual function was assessed using the 19-item FSFI, comprising desire, arousal, lubrication, orgasm, satisfaction, and pain [10]. Standard domain-weighted scoring was used; higher scores indicated better function. The Chinese-language version of the FSFI was used. Women without sexual activity were excluded from the FSFI analysis.

### 2.3. MRI Assessment and Fibroid Classification

Pretreatment pelvic MRI determined dominant fibroid location and whole-uterus morphology. Fibroids predominantly within the anterior uterine wall were classified as anterior; posterior, fundal, lateral, and other locations were classified as non-anterior. Uterine volume, maximal craniocaudal longitudinal diameter, and maximal sagittal anteroposterior diameter were recorded. For each parameter, the 40 women with higher-ranked measurements comprised the larger subgroup and the remaining 35 the smaller subgroup. These sample-defined exploratory divisions were used for exploratory, unadjusted comparisons and do not define clinical cutoffs. The subgroup mean dimensions describe the groups and are not boundary values for classification.

### 2.4. USgHIFU Treatment

USgHIFU was performed using the JC Focused Ultrasound Tumor Therapeutic System (Chongqing Haifu Medical Technology Co., Ltd., Chongqing, China) under real-time ultrasound guidance according to our previously reported protocol [5]. Energy was delivered sequentially to induce coagulative necrosis while minimizing injury to adjacent tissues. Parameters were adjusted according to fibroid depth, anatomical relationships, and real-time echogenic changes.

### 2.5. Outcome Measures and Statistical Analysis

Principal outcomes were baseline-to-6-month changes in OABSS, standardized UDI-6 and IIQ-7, ICIQ-UI SF, and OABSS ≥ 3 prevalence. Urinary changes were also described separately within location groups as exploratory analyses. Exploratory analyses examined associations of the three morphological parameters with baseline OABSS and OABSS ≥ 3 prevalence. Overall FSFI change was secondary.

Continuous variables are presented as mean ± standard deviation and categorical variables as *n* (%). Baseline-to-6-month questionnaire scores were compared using paired-samples *t* tests and OABSS ≥ 3 prevalence using the two-sided exact McNemar test. Location analyses were within-group comparisons and were not interpreted as formal between-group tests of treatment response. Morphological subgroups were compared using Welch’s *t* test for OABSS and Fisher’s exact test for OABSS ≥ 3. Tests were two-sided, with *p* < 0.05 indicating statistical significance. No multiple-comparison adjustment was applied to exploratory subgroup or FSFI-domain analyses. For the baseline morphology comparisons, unadjusted mean differences and 95% confidence intervals were calculated using Welch’s method from the reported group means, standard deviations, and sample sizes. These estimates are approximate because the summary statistics are rounded. Mean changes were expressed as 6-month minus baseline scores; negative values indicate lower symptom scores. Statistical analyses were performed using IBM SPSS Statistics, version 30.0 (IBM Corp., Armonk, NY, USA).

## 3. Results

### 3.1. Participant Characteristics and Female Sexual Function

Among 75 women, 45 (60.0%) had an anterior and 30 (40.0%) had a non-anterior dominant fibroid. Mean age was 39.0 ± 5.7 years, body mass index was 22.6 ± 3.5 kg/m^2^, and pretreatment uterine volume was 630.2 ± 507.1 cm^3^ (Table 1). Among 63 women with paired FSFI assessments, the mean total score increased from 21.6 ± 5.8 to 26.5 ± 6.6 (*p* < 0.005; Table 2).

### 3.2. Overall Urinary Outcomes

At 6 months, all urinary questionnaire scores improved (Table 3). OABSS decreased from 3.51 ± 3.07 to 2.57 ± 2.29 (*p* = 0.005), and OABSS ≥ 3 prevalence decreased from 53.3% (40/75) to 40.0% (30/75; *p* = 0.002). Standardized UDI-6 decreased from 16.07 ± 14.60 to 9.87 ± 11.39 (*p* < 0.001), standardized IIQ-7 from 11.19 ± 17.88 to 4.97 ± 12.61 (*p* < 0.001), and ICIQ-UI SF from 3.01 ± 4.60 to 1.85 ± 3.17 (*p* = 0.009). The corresponding mean changes were −0.94 points for OABSS, −6.20 for standardized UDI-6, −6.22 for standardized IIQ-7, and −1.16 for ICIQ-UI SF. The proportion with OABSS ≥ 3 decreased by 13.3 percentage points.

### 3.3. Urinary Outcomes According to Dominant Fibroid Location

Within the non-anterior group, OABSS (3.60 ± 2.82 vs. 2.70 ± 2.14; *p* = 0.007), standardized UDI-6 (15.17 ± 14.58 vs. 8.33 ± 8.60; *p* = 0.001), and OABSS ≥ 3 prevalence (63.3% vs. 43.3%; *p* = 0.031) decreased; standardized IIQ-7 (*p* = 0.108) and ICIQ-UI SF (*p* = 0.119) did not change significantly (Table 4).

Within the anterior group, standardized UDI-6 (16.67 ± 14.74 vs. 10.89 ± 12.92; *p* = 0.021), standardized IIQ-7 (10.48 ± 15.18 vs. 3.62 ± 10.75; *p* < 0.001), and ICIQ-UI SF (3.11 ± 4.46 vs. 1.80 ± 2.97; *p* = 0.037) decreased. Changes in OABSS (3.44 ± 3.26 vs. 2.49 ± 2.40; *p* = 0.060) and OABSS ≥ 3 prevalence (46.7% vs. 37.8%; *p* = 0.125) were not significant. These were within-group, not between-group, comparisons.

### 3.4. Baseline OAB Symptom Burden According to Uterine Morphology

**Figure 2:**
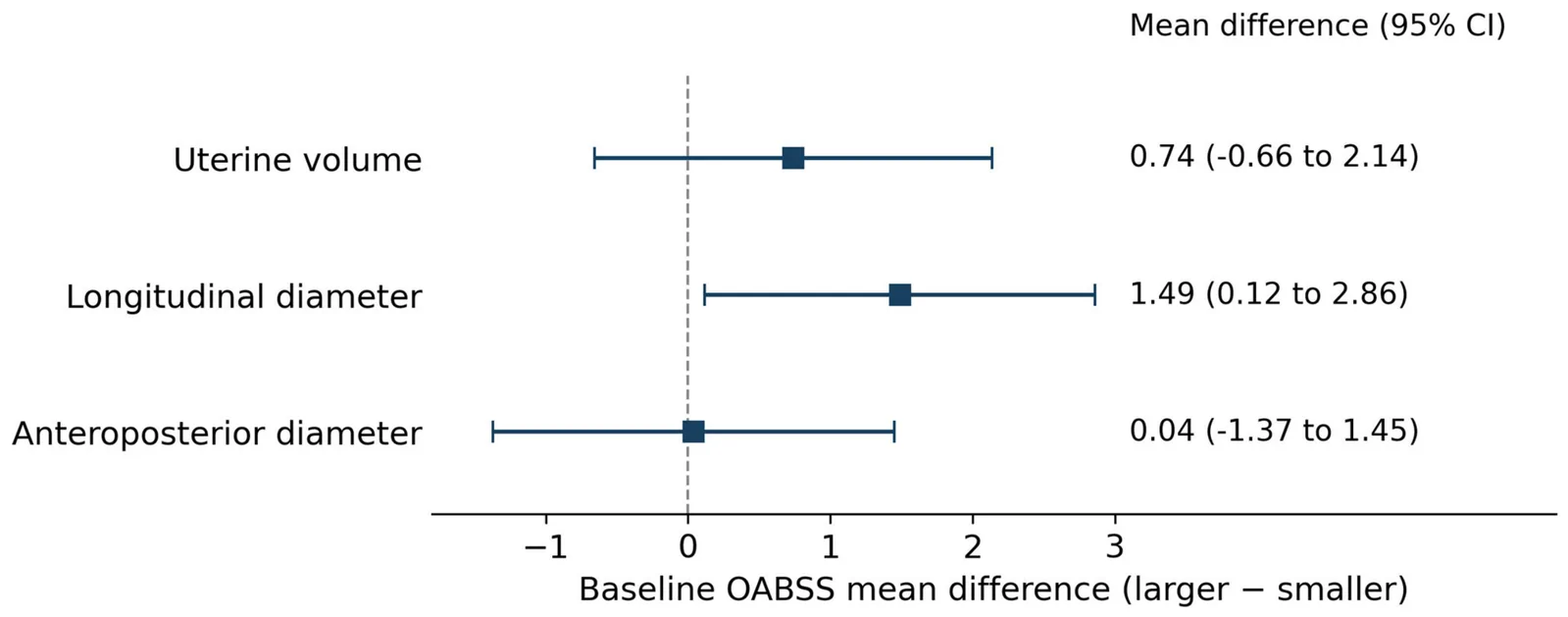
Baseline OABSS mean differences between larger and smaller uterine morphology groups. Squares denote mean differences and horizontal bars denote 95% confidence intervals calculated using Welch’s method from the reported group means, standard deviations, and sample sizes in Table 5 (larger, *n* = 40; smaller, *n* = 35). Positive differences indicate higher baseline OABSS in the larger group. Estimates are approximate because the source summaries are rounded. These unadjusted comparisons concern sample-defined rank groups and do not represent postoperative changes or validated clinical cutoffs. OABSS, Overactive Bladder Symptom Score; CI, confidence interval.

The larger longitudinal-diameter subgroup had higher baseline OABSS (4.20 ± 3.19 vs. 2.71 ± 2.77; *p* = 0.034) and OABSS ≥ 3 prevalence (70.0% vs. 34.3%; *p* = 0.003) (Table 5). Neither outcome differed significantly between larger and smaller subgroups defined by uterine volume (OABSS: *p* = 0.294; OABSS ≥ 3: *p* = 0.252) or anteroposterior diameter (*p* = 0.955 and *p* = 0.819, respectively). The unadjusted OABSS mean differences between larger and smaller subgroups were 0.74 points (95% CI, −0.66 to 2.14) for uterine volume, 1.49 points (95% CI, 0.12 to 2.86) for longitudinal diameter, and 0.04 points (95% CI, −1.37 to 1.45) for anteroposterior diameter (Figure 2).

## 4. Discussion

The principal finding of this study was that urinary symptoms and urinary symptom-related quality of life improved significantly at 6 months after USgHIFU. OABSS, standardized UDI-6 and IIQ-7 scores, and ICIQ-UI SF scores all decreased, together with a reduction in the proportion of women with OABSS ≥ 3. Mean urinary symptom scores decreased in both the anterior and non-anterior fibroid groups. Among the evaluated morphological parameters, a larger longitudinal uterine diameter was associated with greater baseline overactive bladder symptom burden, whereas the corresponding subgroup comparisons for uterine volume and anteroposterior diameter were not statistically significant. Overall female sexual function also improved as a secondary treatment outcome. The mean OABSS reduction was modest (0.94 points from a baseline mean of 3.51), despite statistical significance. This finding reflects a change in average symptom burden, and its clinical relevance at the individual level should be interpreted cautiously.

The overall urinary improvement observed in this study is consistent with previous evidence showing that treatment of uterine fibroids may reduce LUTS and improve health-related quality of life [1]. A systematic review and meta-analysis demonstrated that HIFU was associated with reductions in fibroid volume and symptom severity and with improved quality of life [4]. Similarly, the previous 500-case study from our institution reported improvements in patient-reported urinary symptoms and quality of life after HIFU [5]. Improvements in urinary symptoms have also been reported after surgical treatment of uterine fibroids [11]. A possible explanation is that reduction in fibroid burden decreases pelvic pressure, bladder compression, and anatomical distortion. Nevertheless, the present study did not directly correlate changes in fibroid or uterine dimensions with changes in urinary scores. Therefore, the observed improvement cannot be attributed solely to anatomical shrinkage; improvements in bleeding, pain, sleep disturbance, or overall fibroid-related symptom burden may also have contributed.

The relationship between fibroid location and LUTS remains uncertain. Parker-Autry et al. reported more severe voiding symptoms among women with dominant anterior fibroids, supporting the conventional assumption that an anterior pelvic mass may exert pressure on the bladder or bladder outlet [2]. Berujon et al. similarly found that anterior fibroid location was associated with higher preoperative UDI-6 and ICIQ-UI SF scores [11]. In contrast, Huang et al. found that anterior fibroid position was not consistently associated with urinary incontinence or frequent urination [3]. In the present study, mean urinary symptom scores decreased in both location groups. These within-group findings describe the observed symptom changes but do not establish whether the magnitude of improvement differs according to dominant fibroid location. A direct comparison of changes or a baseline-adjusted analysis would be needed to determine whether fibroid location modifies urinary outcomes after USgHIFU.

The most notable morphological finding was the association between longitudinal uterine diameter and baseline OAB symptom burden. Women in the larger longitudinal-diameter subgroup had higher baseline OABSS and were more likely to have OABSS ≥ 3. Corresponding associations were not observed for total uterine volume or anteroposterior diameter. Previous studies examining the association between anatomical size and LUTS have yielded inconsistent results. Parker-Autry et al. found an association between greater uterine volume and urinary urgency [2], whereas Huang et al. reported that total leiomyoma volume was associated with selected urinary-incontinence outcomes but not with frequent daytime or nighttime urination [3]. In contrast, Bochenska et al. found that neither uterine volume nor fibroid size was associated with pelvic floor symptom burden [12]. Agu et al. reported that leiomyomas larger than 6 cm were associated with LUTS, whereas anterior location and total uterine volume were not [13]. Taken together, these findings indicate that no single measurement of uterine or fibroid size consistently explains urinary symptom burden and support the evaluation of directional uterine dimensions in addition to total uterine volume.

Longitudinal uterine diameter may capture the direction of uterine enlargement rather than overall uterine size alone. Uteri with similar volumes may differ in shape, orientation, and craniocaudal extension within the pelvis. Longitudinal enlargement could therefore alter the position of the bladder or the uterovesical relationship without necessarily producing a greater total uterine volume or anteroposterior diameter. This interpretation remains hypothetical because bladder displacement, uterovesical angle, functional bladder capacity, and pelvic–organ relationships were not directly measured. The association was observed between sample-defined categories and may depend on the grouping method. Whether it persists with continuous measurements and adjustment for age, body mass index, fibroid location, and other clinical factors remains undetermined. These findings do not establish a clinical cutoff or a predictor of urinary improvement after USgHIFU.

Overall female sexual function also improved at 6 months. This finding is consistent with the study by Wang et al., in which sexual function improved after both HIFU and conventional myomectomy [6]. Effective treatment may improve sexual well-being by reducing bleeding, pelvic pressure, pain, or concerns related to fibroid symptoms while preserving the uterus. However, female sexual function is multidimensional and may also be influenced by relationship factors, psychological well-being, body image, and sexual activity. Accordingly, the FSFI findings in the present study should be regarded as a supportive secondary outcome rather than evidence of a specific anatomical mechanism. An increase in the mean FSFI score alone does not establish resolution of sexual dysfunction in individual women.

These findings have several clinical implications. Urinary symptoms should be assessed directly using validated questionnaires before treatment and during follow-up, rather than inferred solely from the location of the dominant fibroid. Women with non-anterior fibroids may still have clinically relevant LUTS and may experience urinary improvement after USgHIFU. Whole-uterus measurements may provide additional information regarding urinary symptom burden, although longitudinal uterine diameter should not yet be considered an established clinical marker or treatment-response predictor. Its potential value should be evaluated using continuous measurements and adjusted statistical models in larger cohorts. The observed score changes may inform counseling about possible urinary symptom benefit; anatomical measurements alone should not be used to predict individual improvement.

Treatment counseling should also consider procedure-specific long-term risks. Disseminated peritoneal leiomyomatosis has been reported five years after laparoscopic supracervical hysterectomy with uncontained morcellation [14]. USgHIFU does not require tissue morcellation, although the present study does not establish comparative long-term safety against surgical alternatives. Diagnostic assessment is also important when selecting patients for USgHIFU. Rare benign variants, such as cotyledonoid dissecting leiomyoma, may have an appearance and growth pattern suggestive of malignancy despite benign histology [15]. Such diagnostic complexity warrants careful assessment of atypical uterine masses before treatment selection.

Several limitations should be acknowledged. First, the retrospective, single-center design, modest sample size, and absence of an untreated control group limit the generalizability of the findings and preclude firm causal inference. Restriction to women with complete paired assessments may introduce selection bias if questionnaire availability is related to symptom response or patient satisfaction. The total screening denominator and reasons for non-inclusion were not established in this analysis, so this potential bias could not be quantified. Second, the location analyses relied on within-group comparisons; differences in statistical significance between groups do not necessarily indicate differences in treatment response. Third, the morphological analyses were exploratory and based on rank-defined subgroups without established clinical cutoffs, continuous-variable analyses, or multivariable adjustment. The multiple comparisons performed may also have increased the possibility of chance findings. Finally, urinary outcomes were based on patient-reported questionnaires without objective urinary assessments or direct correlations between post-treatment anatomical and symptomatic changes, and follow-up was limited to 6 months. The distribution of paired score differences and the confidence intervals for paired changes were not verified from the retained summary results; this limits assessment of test assumptions and the precision of the longitudinal estimates. These considerations should be taken into account when interpreting the subgroup findings. Information on treatment for urinary symptoms, pelvic floor physiotherapy, and hormonal therapy during follow-up was not documented in the study records. These potential confounders may have influenced the observed outcomes.

## 5. Conclusions

USgHIFU was associated with significant improvements in lower urinary tract symptoms, urinary symptom-related quality of life, and overall female sexual function at 6 months. Mean urinary symptom scores decreased in both the anterior and non-anterior fibroid groups. The larger longitudinal-diameter subgroup had greater baseline overactive bladder symptom burden; the corresponding volume and anteroposterior-diameter comparisons were not statistically significant. These exploratory findings support further evaluation of longitudinal uterine diameter in relation to baseline urinary symptom burden, with confirmation in larger prospective studies using continuous measurements and adjusted analyses.

## Figures and Tables

**Table 1:** Demographic data (*n* = 75) are given as mean ± standard deviation or *n* (%).

	Overall (*n* = 75)
Age, years	39.0 ± 5.7
Body mass index, kg/m^2^	22.6 ± 3.5
Uterine volume, cm^3^	630.2 ± 507.1
Follow-up (months)	6

Values are expressed as mean ± standard deviation or numbers.

**Table 2:** Changes in scores of the Female Sexual Function Index (FSFI) before and after treatment with HIFU (*n* = 63).

	Pre-Treatment	Post-Treatment	*p* Value
Desire (1, 2)	3.3 ± 1.4	4.0 ± 1.6	0.03 *
Arousal (3–6)	3.4 ± 0.9	4.6 ± 2.3	0.022 *
Lubrication (7–10)	3.5 ± 2.4	4.8 ± 1.9	0.045 *
Orgasm (11–13)	3.7 ± 1.6	5.0 ± 1.1	<0.002 *^,†^
Satisfaction (14–16)	3.8 ± 1.8	4.0 ± 2.2	0.12
Pain (17–19)	3.9 ± 1.3	4.1 ± 2.3	0.07
FSFI (Total score)	21.6 ± 5.8	26.5 ± 6.6	<0.005 ^†^

* significant difference; data are given as median (range) or mean ± standard deviation. ^†^ Conservative upper bound for the two-sided paired-samples *t*-test *p* value, derived from the reported paired sample size, means, and standard deviations, allowing for rounding.

**Table 3:** Overall changes in urinary outcomes at 6 months after USgHIFU (*n* = 75).

Outcome	Baseline	6 Months	*p* Value
OABSS	3.51 ± 3.07	2.57 ± 2.29	0.005
OABSS ≥ 3, *n* (%)	40 (53.3)	30 (40.0)	0.002
UDI-6	16.07 ± 14.60	9.87 ± 11.39	<0.001
IIQ-7	11.19 ± 17.88	4.97 ± 12.61	<0.001
ICIQ-UI SF	3.01 ± 4.60	1.85 ± 3.17	0.009

Values are presented as mean ± standard deviation unless otherwise indicated. *p* values for continuous outcomes were determined using paired-samples *t* tests. Changes in the proportion of women with OABSS ≥ 3 were assessed using the two-sided exact McNemar test. Paired transition counts are presented in Supplementary Table S1.

**Table 4:** Changes in urinary outcomes according to dominant fibroid location.

Outcome	Non-Anterior (*n* = 30)		*p* Value	Anterior (*n* = 45)		*p* Value
	Baseline	6 Months		Baseline	6 Months	
OABSS	3.60 ± 2.82	2.70 ± 2.14	0.007	3.44 ± 3.26	2.49 ± 2.40	0.060
OABSS ≥ 3, *n* (%)	19 (63.3)	13 (43.3)	0.031	21 (46.7)	17 (37.8)	0.125
UDI-6	15.17 ± 14.58	8.33 ± 8.60	0.001	16.67 ± 14.74	10.89 ± 12.92	0.021
IIQ-7	12.24 ± 21.54	7.00 ± 14.94	0.108	10.48 ± 15.18	3.62 ± 10.75	<0.001
ICIQ-UI SF	2.87 ± 4.88	1.93 ± 3.50	0.119	3.11 ± 4.46	1.80 ± 2.97	0.037

Values are presented as mean ± standard deviation or *n* (%). *p* values for continuous outcomes represent within-group comparisons using paired-samples *t* tests. Changes in the proportion of women with OABSS ≥ 3 were assessed using the two-sided exact McNemar test. Paired transition counts are presented in Supplementary Table S1.

**Table 5:** Baseline overactive bladder symptom burden according to uterine morphology (*n* = 75).

Morphological Parameter	Subgroup	*n*	Morphological Measurement	Baseline OABSS	*p* Value	OABSS ≥ 3, *n* (%)	*p* Value
Uterine volume	Larger	40	936.3 ± 520.3 cm^3^	3.85 ± 3.36	0.294	24 (60.0)	0.252
	Smaller	35	280.5 ± 114.0 cm^3^	3.11 ± 2.70		16 (45.7)	
Longitudinal diameter	Larger	40	135.43 ± 32.21 mm	4.20 ± 3.19	0.034	28 (70.0)	0.003
	Smaller	35	83.58 ± 13.03 mm	2.71 ± 2.77		12 (34.3)	
Anteroposterior diameter	Larger	40	107.30 ± 15.02 mm	3.53 ± 3.33	0.955	22 (55.0)	0.819
	Smaller	35	71.45 ± 12.17 mm	3.49 ± 2.80		18 (51.4)	

Values are presented as mean ± standard deviation or *n* (%). Larger subgroups comprised the 40 women with higher-ranked measurements, whereas smaller subgroups comprised the remaining 35 women. These rank-based divisions were used for exploratory analyses and did not represent clinically validated cutoff values. *p* values were calculated using Welch’s *t* test for continuous OABSS and Fisher’s exact test for OABSS ≥ 3.

## Data Availability

The data presented in this study are available from the corresponding author upon reasonable request, subject to institutional and ethical approval. The data are not publicly available due to patient privacy and ethical restrictions.

## Supplementary Materials

The following supporting information can be downloaded at: https://www.mdpi.com/article/10.3390/biomedicines14102219/s1, Table S1: Transitions across the OABSS threshold from baseline to 6 months.
