# Supplementary material for: Urinary and Sexual Function Outcomes After Ultrasound-Guided High-Intensity Focused Ultrasound for Uterine Fibroids: Associations of Lower Urinary Tract Symptoms with Fibroid Location and Uterine Morphology
Source: Biomedicines. 2026 Oct 1;14(10):2219. doi: 10.3390/biomedicines14102219 (PMC13701314; doi:10.3390/biomedicines14102219)
Supplement: Supplementary file 1 [file biomedicines-14-02219-s001.zip › biomedicines-4592999-supplementary.pdf]

Supplementary Table S1. Transitions across the OABSS threshold from baseline to 6 months

Baseline OABSS	6-month OABSS ≥3	6-month OABSS <3	Baseline total
Overall cohort (n = 75)			
≥3	30	10	40
<3	0	35	35
Non-anterior dominant fibroid (n = 30)			
≥3	13	6	19
<3	0	11	11
Anterior dominant fibroid (n = 45)			
≥3	17	4	21
<3	0	24	24

Two-sided exact McNemar P values: overall, 0.002; non-anterior group, 0.031; anterior group, 0.125.
OABSS, Overactive Bladder Symptom Score. Values are numbers of women. OABSS ≥3 denotes the study-defined symptom-burden threshold, not a definitive diagnosis of overactive bladder.
The discordant pairs were 10 versus 0 overall, 6 versus 0 in the non-anterior group, and 4 versus 0 in the anterior group, where the first count denotes a transition from OABSS ≥3 to <3 and the second denotes the reverse transition. No participant transitioned from OABSS <3 to ≥3.
P values were calculated using the two-sided exact McNemar test, based on the binomial distribution of the discordant pairs under a null probability of 0.5, and are reported to three decimal places. Location-specific P values are within-group comparisons and do not test differences in response between locations.
